# Numerical analysis of unsteady momentum and heat flow of dusty tangent hyperbolic fluid in three dimensions

**DOI:** 10.1038/s41598-022-20457-4

**Published:** 2022-09-27

**Authors:** Madiha Bibi, A. Zeeshan, M. Y. Malik

**Affiliations:** 1 Department of Mathematics, Quaid-i-Azam University, Islamabad, 44000 Pakistan; 2 Department of Mathematics, Purdue University, 150 N. University Street, West Lafayette, IN 47907 USA; 3 Department of Mathematics and statistics, FBAS, International Islamic University, Islamabad, 44000 Pakistan; 4 Department of Mathematics, College of Sciences, King Khalid University, Abha, 61413 Saudi Arabia

**Keywords:** Mathematics and computing, Nanoscience and technology, Physics

## Abstract

This paper explores the impact of MHD and viscous dissipation with joule heating on convective stretching flow of dusty tangent hyperbolic fluid over a sheet in 3D. A time-dependent magnetic field is applied along the z-axis and the sheet being stretched along the xy-plane. The fluid and dust particles motions are coupled only through drag and heat transfer between them. The effect of viscous dissipation with convection is appreciable when the generated kinetic energy becomes appreciable as compared to the amount of heat transferred. A well known bvp4c method has been used to find the fruitful results. Graphs and tables show the facts and figures for physical properties according to different parameters. The main findings are that Increase in power law index, magnetic field, Weissenberg effect, concentration of dust particles, and unsteadiness parameter reduces the flow of fluid and solid granules.

## Introduction

Flow behavior of solid-liquid two-phase flow systems depends on the properties of the dispersed solid phase, the continuous liquid phase that suspends the solids, and the interactions between the two phases. There are many strong reasons of study of fluid dynamics at macro and micro-level as well. At macro level mathematicians and engineers try to investigate the flows without defined boundaries but the case of nano or micro level is really very interesting. In this article we discussed how the surface forces affect the non-Newtonian behavior of solid-liquid suspensions, with the aim of having a deeper understanding of the rheological phenomena.In the classical technologies, paints, coatings, cement slurries, coal slurries, mineral tailings, ceramic oxides, drugs, and food materials are only a few of the many diverse applications. Concentrated suspensions have an immense significance not only in the classical technologies, but also in the emerging technologies as well as in biological systems. We can also detect the presence of a micro-organisms or any impurity in water or in any other liquid. Micro-organism could be considered as particles, not actually but in reference of size, like viruses are nano-sized (20 to about 100 nanometers in size) and bacteria are mostly micro-sized (about 0.5–3 $\mu m$). Such type of study moves towards two phase or multiphase flows. In this write-up dust-particles are submerged in non-Newtonian tangent hyperbolic fluid. The prime-mover for the study of dusty flows was Saffmann^1^. Afterwards Drew^2^ derived a set of coupled equations Orr–Sommerfield, which were helpful to govern the infinitesimal distribution of dust particles in fluid. Then Mekheimer et al.^3^ used the same concept of equations gave results for peristaltic flow in a channel. Recently Bhatti and Zeeshan^4^ discussed the non-Newtonian flow of solid-liquid suspension. Most related and recent studies are given in following references^5,6^.

The boundary layer flow and heat transfer analysis over a stretching flat surface have many applications in industry such as polymer industry, paper production, rolling and manufacturing of sheets and fibers, drawing of plastic film etc. Parenthetically the study of the particulate flow has significant applications for cooling systems, matter separating systems, and purification of crude oil. The beginners of stretching flows were Sakiadis^7^, Crane^8^, Grubka and Bobba^9^. Wang^10^ started the study of 3-D flow over a stretching surface. Takhar and Nath^11^ studied the unsteady three dimensional flow because of stretching surface. A remarkable role in this research area played by Ariel^12–14^. Latest study regarding three dimensional boundary layer flow is done by Hayat et al.^15^ and Mair et al.^16^.

The consumption of non-Newtonian fluids found in many engineering and industrial processes, such as food mixing, blood flow, mercury amalgams, and lubrications. In view of intense need, many studies are focused on non-Newtonian fluids. This study is also for the non-Newtonian tangent hyperbolic considered as base fluid which obeys the power law model and capable of describing the shear thinning effects. In the last going decade the boundary layer flow as well as the peristaltic flow considered in the following literature. Akbar et al.^17^ discussed the stretching problem for 2D, Malik et al.^18^ proposed the numerical scheme for the flow over stretching cylinder, and Bibi et al.^19^ investigate the dusty flow over stretching surface. Flow can be disturbed or facilitate by changing the values of different physical parameters. Here we have considered the MHD, effect, joule heating and viscous dissipation with convective boundary conditions. Kumar et al.^20^ discussed the MhD flow of dusty tangent hyperbolic fluid under the effect of thermal radiations. Historically the front runner of the study of viscous dissipation in natural convection was Gebhart^21^. Joule heating (also related to resistive or ohmic heating) is the process where the power of an electric current is converted into heat as it flows through a resistance. The effect of viscous dissipation in natural convection is appreciable when the induced kinetic energy becomes appreciable compared to the amount of heat transferred. Above effects are discussed in a combine way in following different references^22–29^.

Precisely the focus of the current article is to study the three dimensional dusty tangent hyperbolic fluid flow with change in time while considering MHD. This study is different from the previous atricle^30^ in that respect, we have used different non-Newtonain base fluid and estimated heat flow is executed by joule heating and viscous dissipation with convection. The solution of highly non-linear problem is sorted out numerically.

## Modeling

**Figure 1:**
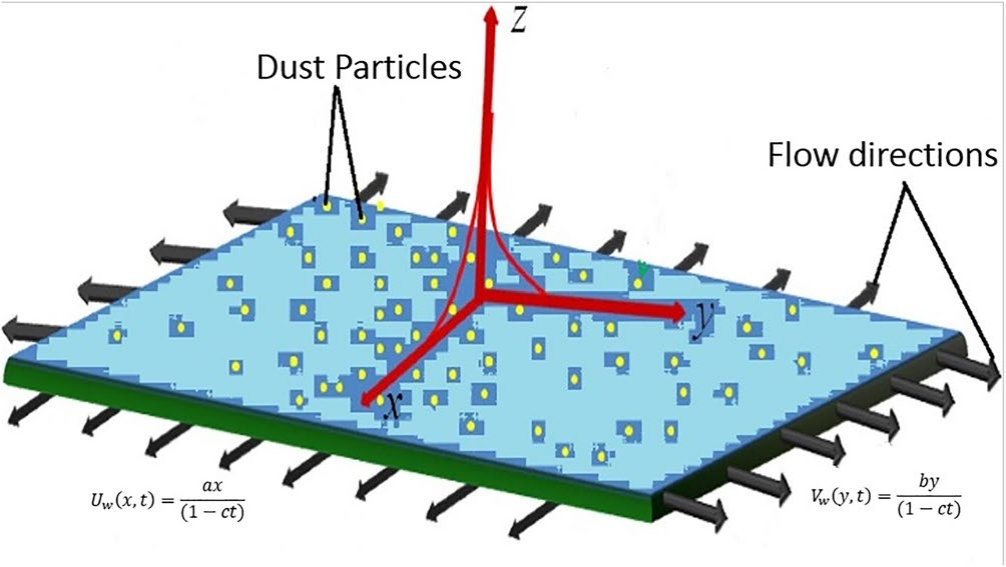
Physical configuration.

The incompressible unsteady dusty tangent hyperbolic non-Newtonian fluid flow is considered in trheedimenional space. The consideration of temperature profile is under the effect of MHD (Magnetohydrodynamic), Joule heating and viscous dissipation with convective boundary conditions. Flow distribution is due to stretching sheet which is assumed to be placed in xy-plane and fluid is placed along the z-axis. The physical configuration is given in Fig. 1. The two phase modeling is followed by the reference^4^ and the cauchy stress tensor for tangent hyperbolic fluid is mentioned in the reference^19^,

1 $$\begin{aligned} \tau =[\mu _\infty +(\mu _\circ +\mu _\infty )\tanh (\Gamma \dot{\gamma })^n]\dot{\gamma }, \end{aligned}$$

where $\mu _\infty$ is the infinite share rate viscosity and $\mu _\circ$ is the zero rate viscosity of the fluid. *n* is known as power law index, $\Gamma$ is the time constant and $\dot{\gamma }$ is defined below,

2 $$\begin{aligned} \dot{\gamma }=\sqrt{\frac{1}{2}trac(A_1^2)}. \end{aligned}$$

Here $A_1$ is the first Rivilin–Ereckson tensor. The fluid tangent Hyperbolic is shear thinning so the $\Gamma \dot{\gamma }<<1$ should be considered. The tangent hyperbolic series Eq. (1) reduces to,

3 $$\begin{aligned} \tau =\mu _\circ [1+n(\Gamma \dot{\gamma }-1)]\dot{\gamma } \end{aligned}$$

Problem can be modeled as:

4 $$\begin{aligned}&\frac{\partial u}{\partial x}+\frac{\partial v}{\partial y}+\frac{\partial w}{\partial z} = 0, \end{aligned}$$

5 $$\begin{aligned}&\frac{\partial u_P}{\partial x}+\frac{\partial v_P}{\partial y}+\frac{\partial w_p}{\partial z} = 0, \end{aligned}$$

6 $$\begin{aligned}\rho (1-C)\left[ \frac{\partial u}{\partial t}+u\frac{\partial u}{\partial x}+v\frac{\partial u}{\partial y}+w\frac{\partial u}{\partial z}\right]& = \mu (1-C)\frac{\partial }{\partial z}\left[ \left( (1-n)+\sqrt{2} n \Gamma \sqrt{\left( \frac{\partial u}{\partial z}\right) ^2 + \left( \frac{\partial v}{\partial z}\right) ^2}\right) \frac{\partial u}{\partial z}\right] \nonumber \\&\quad -\sigma B^2(t) u + CS(u_{p}-u), \end{aligned}$$

7 $$\begin{aligned}&C\rho _P\left( \frac{\partial u_P}{\partial t}+u_P \frac{\partial u_P}{\partial t}+v_P\frac{\partial u_P}{\partial y}+w_p\frac{\partial u_p}{\partial z}\right) = CS(u-u_P),\;\;\;\; \end{aligned}$$

8 $$\begin{aligned}\rho (1-C)\left[ \frac{\partial v}{\partial t}+u\frac{\partial v}{\partial x}+v\frac{\partial v}{\partial y}+w\frac{\partial v}{\partial z}\right] &=\mu (1-C)\frac{\partial }{\partial z} \left[ \left( (1-n)+\sqrt{2} n \Gamma \sqrt{(\frac{\partial u}{\partial z})^2 + (\frac{\partial v}{\partial z})^2}\right) \frac{\partial v}{\partial z}\right] \nonumber \\&\quad -\sigma B^2(t) v + CS(v_{p}-v), \end{aligned}$$

9 $$\begin{aligned}&C\rho _P\left( \frac{\partial v_P}{\partial t}+u_P \frac{\partial v_P}{\partial t}+v_P\frac{\partial v_P}{\partial y}+w_p\frac{\partial v_p}{\partial z}\right) = CS(v-v_P),\;\;\;\; \end{aligned}$$

10 $$\begin{aligned}\rho c_p(1-C)\left[ \frac{\partial T}{\partial t}+u\frac{\partial T}{\partial x}+v\frac{\partial T}{\partial y}+w\frac{\partial T}{\partial z}\right] &= k(1-C)\frac{\partial ^2 T}{\partial z^2}+\frac{\rho _p c_p C}{\tau _{t}}(T_{P}-T)+CS[(u-u_P)^2\nonumber \\&\quad + (v-v_p)^2]-\mu \left[ (\frac{\partial u}{\partial z})^2+\left( \frac{\partial v}{\partial z}\right) ^2\right] +\sigma B^2(u^2 +v^2) \end{aligned}$$

11 $$\begin{aligned}&C\left( \frac{\partial T_P}{\partial t}+u_P\frac{\partial T_P}{\partial x}+v_P\frac{\partial T_P}{\partial y}+w_p\frac{T_p}{z}\right) = C\frac{c_P}{c_m \tau _T}(T-T_P), \end{aligned}$$

conditions at the boundary are:

12 $$\begin{aligned} u=U_w(x,t), \; v=V_w(y,t),\;w=0,\; -k\frac{\partial T}{\partial z}=h_f(T_w-T), \;\;at\; z=0,\;\;\;\;\;\;\;\;\;\;\;\;\;\; \end{aligned}$$

13 $$\begin{aligned} u_P=u= 0,\; v_P=v=0,\; w_p=w,\; T\rightarrow T_{\infty },\; T_P\rightarrow T_{\infty }\; as \; z\rightarrow \infty .\;\;\;\;\;\;\; \end{aligned}$$

*u*, *v* and *w* are the fluid velocity components in abscissa, ordinate and applicate axis direction. Similarly $u_P$, $v_P$ and $w_p$ are particles velocity components. $\rho$ is the density of the fluid and $\rho _P$ is the density of the particles. $\sigma$ is for electrical conduction. *B*(*t*) is the magnetic field. *T* is the fluid temperature and $T_P$ is temperature of particles respectively. $U_w$ and $V_w$ are the stretching velocities. $T_w$ is the temperature of wall, $h_f$ is the heat transfer coefficient (HTC) and *k* is thermal diffusion. *C* is the volume fraction of the solid particles, *S* is the drag force, there are different correlations of it according to assumptions are defined by Chhabra in his book^31^. Here the considered value for *S* is given below,

14 $$\begin{aligned} S=\frac{9}{2}\frac{\mu _\circ }{r^2}\lambda (C), \end{aligned}$$

15 $$\begin{aligned} \lambda (C)=\frac{4+3[8C-3C^2]^{1/2} +3C}{[2-3C]^2}. \end{aligned}$$

Value of the above function is determined by Tam^32^. The correlation for viscosity of fluid-particle mixture is proposed by Charm and Kurland^33^.

16 $$\begin{aligned} \mu _d=\mu _\circ \frac{1}{1-qC}, \end{aligned}$$

17 $$\begin{aligned} q=0.07 exp[2.49C+\frac{1107}{T}exp(-1.69C)]. \end{aligned}$$

For the conversion of Partial Differential Equations into Ordinary Differential Equations one need some transformations. Similarity transformations required for the conversion of PDE’s to ODE’s can be defined as

18 $$\begin{aligned} u=\frac{ax}{1-ct}f'(\eta ),\; v=\frac{ay}{1-ct}g'(\eta ),\;w=-\sqrt{\frac{\nu a}{1-ct}}(f(\eta )+g(\eta )),\; \theta =\frac{T-T_\infty }{T_w-T_\infty },\nonumber \\ u_P=\frac{ax}{1-ct}F'(\eta ),\; v_P=\frac{ay}{1-ct}G'(\eta ),\; w_P=-\sqrt{\frac{\nu a}{1-ct}}(F(\eta )+G(\eta )),\; \theta _P=\frac{T_P-T_\infty }{T_w-T_\infty }, \end{aligned}$$

and to be noted that,

19 $$\begin{aligned} U_w(x,t)=\frac{ax}{1-ct},\; V_w(y,t)=\frac{by}{1-ct},\;\eta =z\sqrt{\frac{U_w}{\nu x}},\; T_w(x,t)=T_\infty +\frac{T_o U_w x}{\nu (1-ct)^\frac{1}{2}},\;B(t)=\frac{B_o}{(1-ct)^\frac{1}{ 2}}. \end{aligned}$$

Here *c* is a balancing constant and $a>0$ is for accelerated flows and $B_o$ is the amplitude of applied magnetism. Using Eqs. (18)–(19) into Eqs. (4)–(13),

20 $$\begin{aligned} & f^{{\prime \prime \prime }} \left[ {(1 - n) + nWe_{x} \left( {\sqrt {f^{{\prime 2}} + g^{{\prime 2}} } + \frac{{f^{{\prime \prime 2}} }}{{\sqrt {f^{{\prime 2}} + g^{{\prime 2}} } }}} \right)} \right] + nWe_{x} \frac{{f^{{\prime \prime }} g^{{\prime \prime }} g^{{\prime \prime \prime }} }}{{\sqrt {f^{{\prime 2}} + g^{{\prime 2}} } }} + (f + g)f^{{\prime \prime }} - f^{{\prime 2}} - A[f^{\prime } \quad \\ & \quad + \frac{\eta }{2}f^{{\prime \prime }} ] - \frac{{M^{2} }}{{(1 - C)}}f^{\prime } + \frac{{CR}}{{(1 - C)}}(F^{\prime } (\eta ) - f^{\prime } (\eta )) = 0,{\text{ }} \\ \end{aligned}$$

21 $$\frac{A}{2}\eta F^{{\prime \prime }} + AF^{\prime } + F^{{\prime 2}} - (F + G)F^{{\prime \prime }} + R(F^{\prime } - f^{\prime } ) = 0,$$

22 $$\begin{aligned} & g^{{\prime \prime \prime }} \left[ {(1 - n) + nWe_{y} \left( {\sqrt {f^{{\prime 2}} + g^{{\prime 2}} } + \frac{{g^{{\prime \prime 2}} }}{{\sqrt {f^{{\prime 2}} + g^{{\prime 2}} } }}} \right)} \right] + nWe_{y} \frac{{g^{{\prime \prime }} f^{{\prime \prime }} f^{{\prime \prime \prime }} }}{{\sqrt {f^{{\prime 2}} + g^{{\prime 2}} } }} + (f + g)g^{{\prime \prime }} - g^{{\prime 2}} - A[g^{\prime } \quad \\ & \quad + \frac{\eta }{2}g^{{\prime \prime }} ] - \frac{{M^{2} }}{{(1 - C)}}g^{\prime } + \frac{{CR}}{{(1 - C)}}(G^{\prime } (\eta ) - g^{\prime } (\eta )) = 0, \\ \end{aligned}$$

23 $$\frac{A}{2}\eta G^{{\prime \prime }} + AG^{\prime } + G^{{\prime 2}} - (F + G)G^{{\prime \prime }} + R(G^{\prime } - g^{\prime } ) = 0,$$

24 $$\begin{aligned} & \theta ^{{\prime \prime }} + Pr(f\theta ^{\prime } - 2f^{\prime } \theta ) - Pr\frac{A}{2}(\eta \theta ^{\prime } + 3\theta ) + Pr\theta ^{\prime } (f + g) + \frac{{Pr\alpha \beta _{T} C}}{{(1 - C)}}(\theta _{P} - \theta ) \\ & {\kern 1pt} + \frac{{CEc^{*} }}{{(1 - C)}}(f^{\prime } - F^{\prime } )^{2} + \frac{{CEc^{*} }}{{(1 - C)}}(g^{\prime } - G^{\prime } )^{2} + \frac{{Ec_{x} f^{{\prime \prime 2}} }}{{(1 - C)}} + \frac{{Ec_{y} g^{{\prime \prime 2}} }}{{(1 - C)}} + \frac{{M^{2} Ec_{x} f^{{\prime 2}} }}{{(1 - C)}} + \frac{{M^{2} Ec_{y} g^{{\prime 2}} }}{{(1 - C)}} = 0,{\text{ }} \\ \end{aligned}$$

25 $$\theta _P'\left( \eta \frac{A}{2}-2F-G\right) -\gamma \beta _T(\theta -\theta _P)+\theta _P\left( \frac{3}{2}A+2f'\right) =0,$$

along with the boundary conditions

26 $$\begin{aligned} f(0)=0,\; f'(0)=1,\;g(0)=0,\;g'(0)=s,\;\theta '(0)=-\zeta [1-\theta (0)], \;\;\;\;\;\;\;\;\;\;\; \end{aligned}$$

27 $$\begin{aligned} f'\rightarrow 0,\;\;\;\;F'\rightarrow 0,\;\;F=f,\; g'\rightarrow 0,\; G' \rightarrow 0,\; G=g,\;\theta \rightarrow 0,\;\;\;\theta _P\rightarrow 0\;\;\;as\;\;\;\eta \rightarrow \infty .\;\;\;\; \end{aligned}$$

*A* is the unsteadiness parameter, *M* is the magnetic parameter, $We_x$ and $We_y$ are Weissenberg numbers along x and y-direction, *Pr* is Prandtl number, *R* is the fluid-particle interaction for velocity profile, $\beta _T$ is fluid-particle interaction for temperature profile, *C* is the volume fraction of the granules, $\alpha$ is the mass concentration, $\gamma$ is the ratio of the specific heat capacity of the fluid to the particles, *s* is stretching ratio, $Ec^*$ is viscous dissipation parameter and $Ec_x$, $Ec_y$ are Eckert numbers in x and y-direction, defined below

28 $$\begin{aligned} We_x=\sqrt{\frac{2 a^3 \Gamma x^2}{\nu (1-ct)^3}},\; We_y=\sqrt{\frac{2 a^3 \Gamma y^2}{\nu (1-ct)^3}},\; A=\frac{c}{a}, \; M=\sqrt{\frac{\sigma }{\rho a}}B_o,\;\; \nonumber \\ Pr=\frac{\mu C_p}{k}, \; R=S\frac{(1-ct)}{\rho a},\; \alpha =\frac{\rho _P}{\rho }, \gamma =\frac{c_p}{c_m},\; \beta _T=\frac{1-ct}{a\tau _T},\;s=\frac{b}{a},\; \nonumber \\ Ec^*=\frac{S\nu ^2(1-ct)^2}{T_{o} k},\;Ec_x=\frac{U_w^2}{c_p(T_f-T_\infty )},\;Ec_y=\frac{V_w^2}{c_p(T_f-T_\infty )}. \end{aligned}$$

where $\tau _T$ is the equilibrium time, required by dust particles to manage their temperature compatible to fluid. Skin friction for three dimensional flow is mentioned in Eq. (29),

29 $$\begin{aligned} C_{fx}=\frac{\tau _{xz}}{\frac{1}{2}\rho U_w ^2},\; C_{fy}=\frac{\tau _{yz}}{\frac{1}{2}\rho V_w ^2},\;, \end{aligned}$$

coefficients will be reduced to

30 $$\begin{aligned} \tau _{xz}=\mu _o \left[ (1-n)\frac{\partial u}{\partial z}+n\left( \frac{\Gamma }{\sqrt{2}}\sqrt{\frac{\partial u}{\partial z}^2+\frac{\partial v}{\partial z}^2}\right) \frac{\partial u}{\partial z}\right] _{z = 0}, \nonumber \\ \tau _{yz}=\mu _o \left[ (1-n)\frac{\partial v}{\partial z}+n\left( \frac{\Gamma }{\sqrt{2}}\sqrt{\frac{\partial u}{\partial z}^2+\frac{\partial v}{\partial z}^2}\right) \frac{\partial v}{\partial z}\right] _{z = 0}, \end{aligned}$$

by inserting Eq. (30), into Eq. (29), one can get,

31 $$\begin{aligned} C_{fx} Re_x^{\frac{1}{2}}=(1-n)f''(0)+n\frac{We_x}{2}\sqrt{f''(0)^2+g''(0)^2}f''^{2}(0), \end{aligned}$$

32 $$\begin{aligned} C_{fy} Re_y^{\frac{1}{2}}=(1-n)g''(0)+n\frac{ We_y}{2}\sqrt{f''(0)^2+g''(0)^2}g''^{2}(0). \end{aligned}$$

The expression for the Nusselt number is given in Eq. (33),

33 $$\begin{aligned} Nu_{x}=\frac{xq_w}{k(T_w-T_\infty )},\;q_w=-k\left( \frac{\partial T}{\partial z}\right) \end{aligned}$$

where $q_w$ denotes the heat flux of the surface. Settle the value of $q_w$ into $Nu_x$ while considering the thermal radiations effective, one can get following relation for Nusselt number,

34 $$\begin{aligned} Nu_{x}Re_{x}^{-\frac{1}{2}}=-\theta '(0)\;\;\;\;. \end{aligned}$$

Here $Re_x=\frac{U_w x}{\nu }$ and $Re_y=\frac{V_w y}{\nu }$ are the Reynolds numbers.

## Method of solution

The suitable method for the problem is bvp4c. For this one can convert equations in he following form:

35 $$\begin{aligned} f'''&= \frac{1}{\left[ (1-n)+n We_x \left( \sqrt{f'^2 +g'^2}+\frac{f''^2}{\sqrt{f'^2 +g'^2}}\right) \right] } \left[ - n We_x \frac{f'' g'' g'''}{\sqrt{f'^2 +g'^2}}+f'^2+A\left( f'+\frac{\eta }{2}f''\right) \right. \nonumber \\&\quad \left. +\frac{M^2 f'}{1-C}-\frac{C R}{1-C}(F'-f')-f''(f+g)\right] , \end{aligned}$$

36 $$F^{{\prime \prime }} = \frac{{ - AF^{\prime } - F^{{\prime 2}} - R(F^{\prime } - f^{\prime } )}}{{\frac{{A\eta }}{2} - 2F - G}},$$

37 $$\begin{aligned} g'''&= \frac{1}{\left[ (1-n)+ n We_y \left( \sqrt{f'^2 +g'^2}+\frac{g''^2}{\sqrt{f'^2 +g'^2}}\right) \right] }\left[ - n We_x \frac{g'' f'' f'''}{\sqrt{f'^2 +g'^2}}+g'^2+A\left( g'+\frac{\eta }{2}g''\right) \right. \nonumber \\&\quad \left. +\frac{M^2 g'}{1-C}-\frac{C R}{1-C}(G'-g')-g''(f+g)\right] , \end{aligned}$$

38 $$G'' = \frac{-A G'-s G'^2-R(G'-g')}{\frac{A \eta }{2}-F-G},$$

39 $$\begin{aligned} \theta ''&=Pr\frac{A}{2}(\eta \theta '+3\theta )-Pr(f\theta '-2f'\theta ) -\frac{Pr\alpha \beta _T C}{1-C}(\theta _P-\theta )-Pr \theta '(f+g)\nonumber \\&\quad +Ec_x f''^2+Ec_y g''^2 +M^2 Ec_x f'^2 + M^2 Ec_y g'^2-\frac{C Ec^*}{(1-C)}(f'-F')^2-\frac{C Ec^*}{1-C}(g'-G')^2, \end{aligned}$$

40 $$\theta _P'= \frac{\gamma \beta _T(\theta -\theta _P)-\theta _P(\frac{3A}{2}+2F')}{\frac{A\eta }{2}-2F-G}.$$

The required dummy variables as shown in Eq. (41).

41 $$\begin{aligned} f = & y_{1} ,\;f^{\prime} = y_{2} ,\;f^{\prime\prime} = y_{3} ,\;f^{\prime\prime\prime} = y^{\prime}_{3} , \\ F = & y_{4} ,\;F^{\prime} = y_{5} ,\;F^{\prime\prime} = y^{\prime}_{5} , \\ g = & y_{6} ,\;g^{\prime} = y_{7} ,\;g^{\prime\prime} = y8,\;g^{\prime\prime\prime} = y^{\prime}_{8} , \\ G = & y_{9} ,\;G^{\prime} = y_{{10}} ,\;G^{\prime\prime} = y^{\prime}10, \\ \theta = & y_{{11}} ,\;\theta ^{\prime} = y_{{12}} ,\;\theta ^{\prime\prime} = y^{\prime}_{{12}} , \\ \theta _{P} = & y_{{13}} ,\;\theta _{{P^{\prime}}} = y^{\prime}_{{13}} . \\ \end{aligned}$$

Set of Eqs. (35)–(40) can be molded in the initial value problem as:

42 $$\begin{aligned}&\frac{dy_{1}}{dx} = y_{2}, \end{aligned}$$

43 $$\begin{aligned}&\frac{dy_{2}}{dx} = y_{3}, \end{aligned}$$

44 $$\begin{aligned} \frac{dy_{3}}{dx}&= \frac{1}{\left[ (1-n)+ n We_x \left( \sqrt{y_{2}^2 +y_{7}^2}+\frac{y_{3}^2}{\sqrt{f_{2}^2 +y_{7}^2}}\right) \right] }\left[ - n We_x \frac{y_{3} y_{8} y_{8}'}{\sqrt{y_{2}^2 +y_{7}^2}}+y_{2}^2-y_{3}(y_{1}+y_{6})+A(y_{2} \right. \nonumber \\&\quad \left. +\frac{\eta }{2}y_{3})+\frac{M^2}{1-C}y_{2}-\frac{C R}{(1-C)}(y_{5}-y_{2})\right] ,\;\;\; \end{aligned}$$

45 $$\begin{aligned}&\frac{dy_{4}}{dx} = y_{5}, \end{aligned}$$

46 $$\begin{aligned}&\frac{dy_{5}}{dx} = \frac{-A y_{5}-y_{5}^2-R(y_{5}-y_{2})}{\frac{A \eta }{2}-2y_{4}}-y_{9}, \end{aligned}$$

47 $$\begin{aligned}&\frac{dy_{6}}{dx} = y_{7}, \end{aligned}$$

48 $$\begin{aligned}&\frac{dy_{7}}{dx} = y_{8}, \end{aligned}$$

49 $$\begin{aligned} \frac{dy_{8}}{dx}&= \frac{1}{\left[ (1-n)+ n We_y \left( \sqrt{y_{2}^2 +y_{7}^2}+\frac{y_{8}^2}{\sqrt{y_{2}^2 +y_{7}^2}}\right) \right] } \left[ - n We_x \frac{y_{8} y_{3} y_{3}'}{\sqrt{y_{2}^2 +y_{7}^2}}+ y_{7}^2-y_{8}(y_{1}+y_{6})+A(y_{7} \right. \nonumber \\&\quad \left. +\frac{\eta }{2}y_{8})+\frac{M^2}{1-C}y_{7}-\frac{C R}{(1-C)}(y_{10}-y_{7})\right] ,\;\;\; \end{aligned}$$

50 $$\begin{aligned}&\frac{dy_{9}}{dx} = y_{10}, \end{aligned}$$

51 $$\begin{aligned}&\frac{dy_{10}}{dx} = \frac{-A y_{10}-s y_{10}^2-R(y_{10}-y_{7})}{\frac{A \eta }{2}-y_{4}}-y_{9}, \end{aligned}$$

52 $$\begin{aligned}&\frac{dy_{11}}{dx} = y_{12}, \end{aligned}$$

53 $$\begin{aligned} \frac{dy_{12}}{dx}&=-Pr(y_{1}y_{12}-2y_{2}y_{11})+Pr\frac{A}{2}(\eta y_{12}+3y_{11})-\frac{Pr\alpha \beta _T C}{1-C}(y_{13}-y_{11})-Pr y_{12}(y_{1}+y_{6})\nonumber \\&\quad +Ec_x y_{3}^2+Ec_y y_{8}^2 +M^2 Ec_x y_{2}^2 + M^2 Ec_y y_{7}^2-\frac{C Ec^*}{(1-C)}(y_{2}-y_{5})^2-\frac{C Ec^*}{(1-C)}(y_{7}-y_{10})^2, \end{aligned}$$

54 $$\begin{aligned}&\frac{dy_{13}}{dx}=\frac{\gamma \beta _T(y_{11}-y_{13})-y_{13}(\frac{3A}{2}+2 y_{5})}{\frac{A\eta }{2}-2y_{4}-y_{9}}, \end{aligned}$$

the reduced endpoint conditions are

55 $$\begin{aligned}&y_{1}(a)=0,\;y_{2}(a)=1,\;y_{2}(b)=s_{1}, \; y_{4}(b)=y_{1}(b),\nonumber \\&y_{5}(b)=s_{2},\; y_{6}(a)=0,\; y_{7}(a)=s, \; y_{7}(b)=s_{3}, \nonumber \\&y_{9}(b)=y_{6}(b), \; y_{10}(b)=s_{4}, \; y_{11}(b)=s_{5}, \nonumber \\&y_{12}(a)=-\zeta (1-y_{11}(a)), \;y_{13}(b)=s_{6}. \end{aligned}$$

There is need of some initial guesses that are $s_{1},s_{2},s_{3},s_{4},s_{5}$ and $s_{6}$ in a way like integration of the system of ODEs fulfil the conditions at endpoints and obtained the solution for the system of Eq. (42)–(54).

## Results and discussions

**Figure 2:**
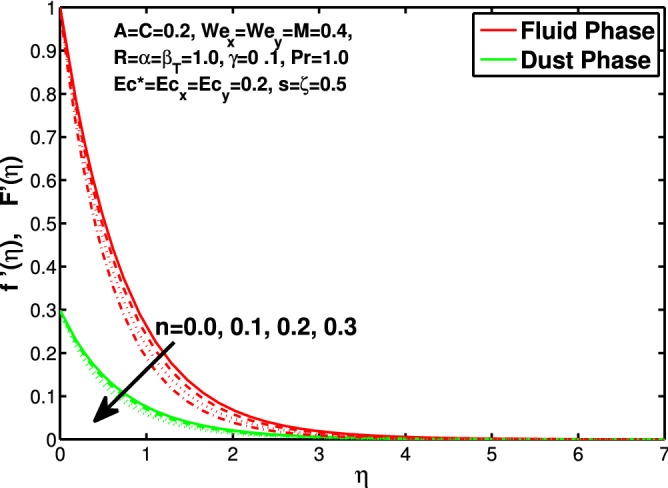
Effect of “*n*” on the velocity distribution in x-direction.

**Figure 3:**
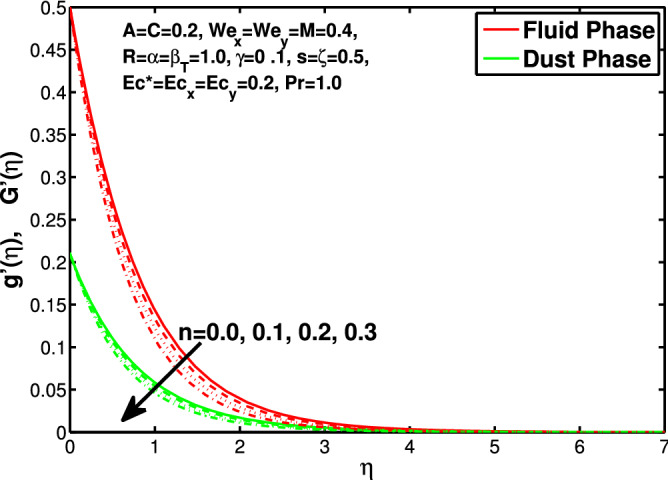
Effect of “*n*” on the velocity distribution in y-direction.

**Figure 4:**
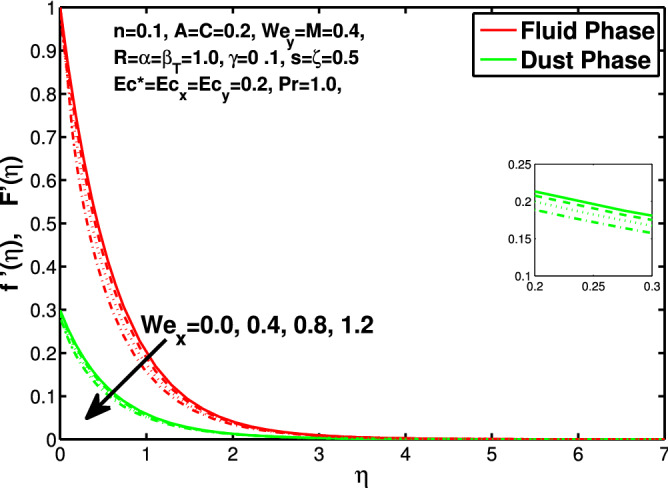
Effect of “$We_x$” on the velocity distribution in x-direction.

**Figure 5:**
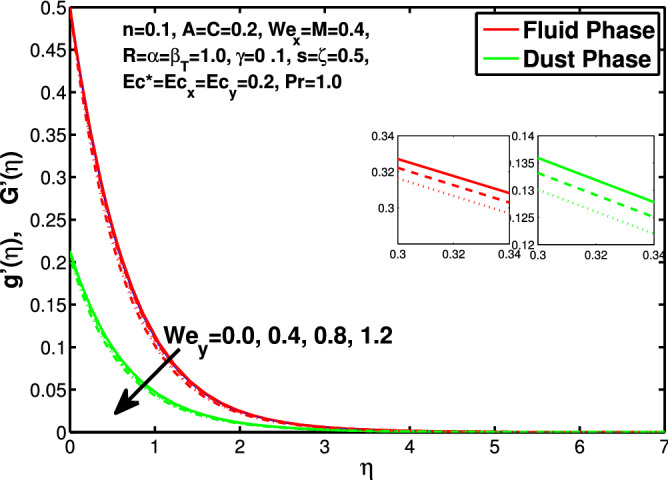
Effect of “$We_y$” on the velocity distribution in y-direction.

**Figure 6:**
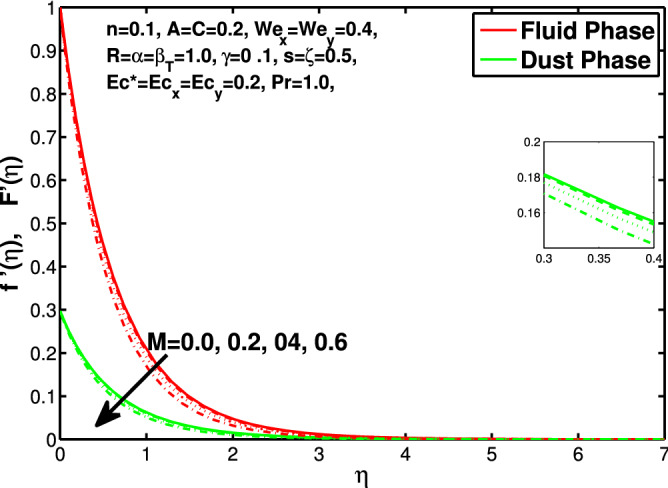
Effect of “*M*” on the velocity distribution in x-direction.

**Figure 7:**
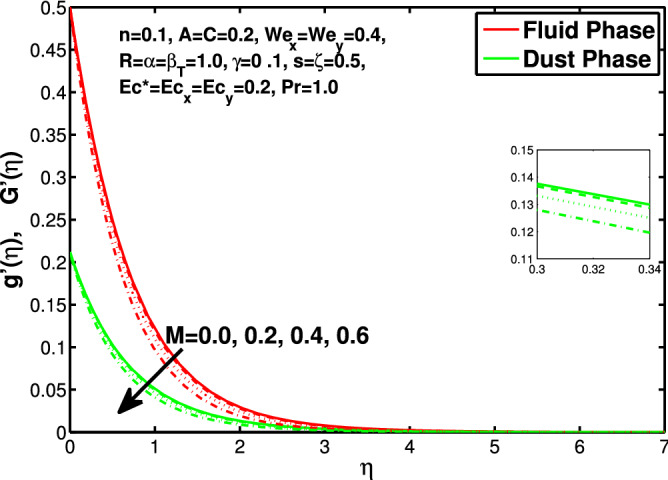
Effect of “*M*” on the velocity distribution in y-direction.

**Figure 8:**
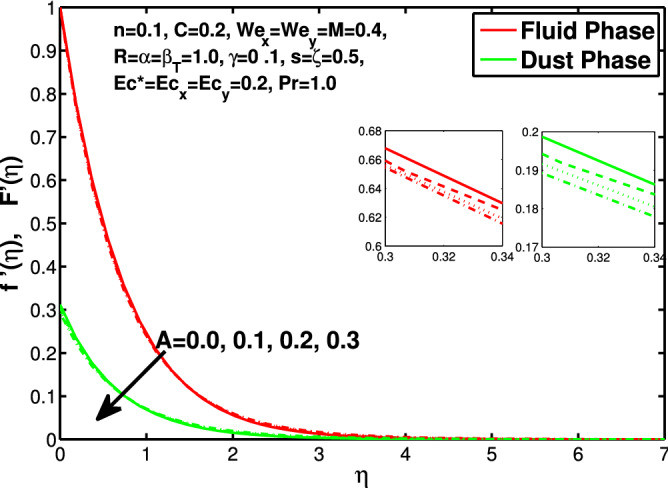
Effect of “*A*” on the velocity distribution in x-direction.

**Figure 9:**
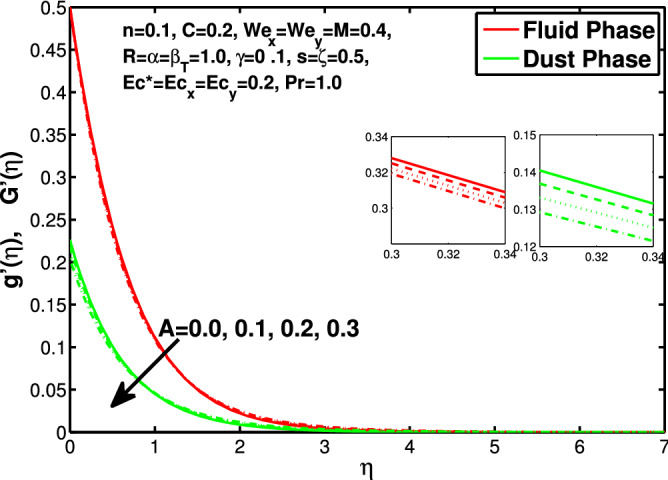
Effect of “*A*’ on the velocity distribution in y-direction.

In this section the results of the momentum and temperature boundary layers discussed graphically and numerically. Figures  2 and 3 show the behavior of the power-law index n that the rise in flow index decreases the velocity of both fluid and granules in both directions. Here the values are checked for $n<1$ which are applicable for pseudoplastics fluids, so we can use the results for shear thinning fluids. Figures 4 and 5 are for describing the Weissenberg effect, $We_x$ and $We_y$ are time dependent parameters. Increase in Weissenberg parameter reduces retardation time will reduce the velocity of fluid and solid particles. The Weissenberg effec is actually rod climbing efffect which is associated with the non-Newtonain flows, so one can use the results for the different non-Newtonian fluids. Figures 6 and 7 shows the effects of applied magnetic field. The magnetic field causes Lorentz force which create hurdle in the fluid flow because of resistive nature, in result of which decreases the flow of fluid and solid particles as well in both directions. Figures 8 and 9 are plotted to check the change in velocity profile due to change in *A*. Graphs show that the increase in unsteadiness, reduces the velocity of fluid and solid particles in both directions. One can see in the graph that velocity decreases near the surface and increases away from surface. As *A* is defined as inversely proportional to stretching coefficient *a*. The increase in unsteadiness parameter *A* reduces the *a*, in result of which velocity of fluid and solid particles decreases.

**Figure 10:**
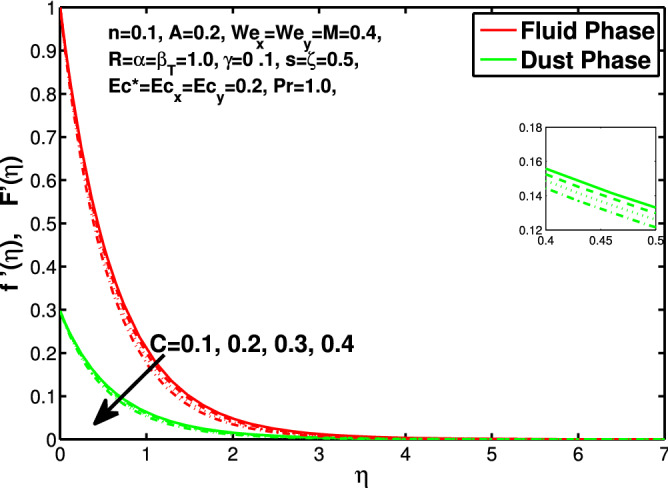
Effect of “*C*” on the velocity distribution in x-direction.

**Figure 11:**
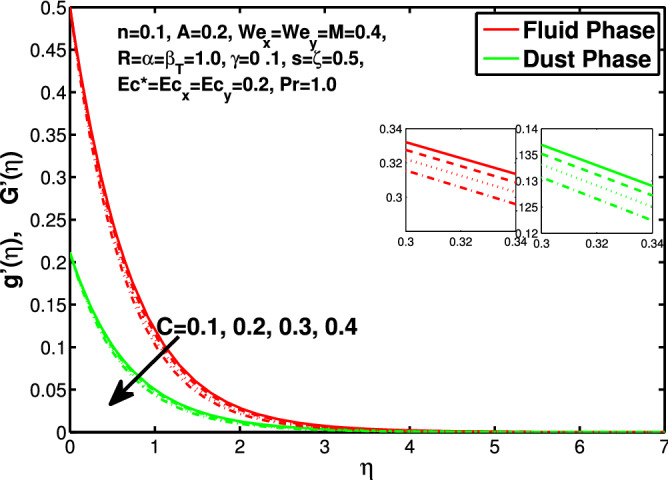
Effect of “*C*” on the velocity distribution in y-direction.

**Figure 12:**
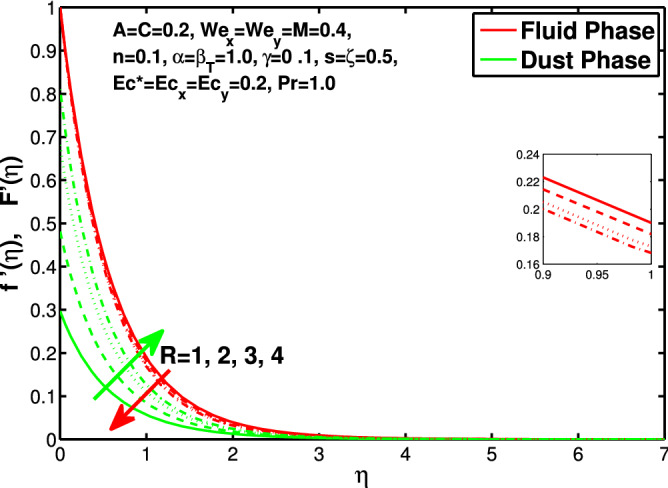
Effect of “*R*” on the velocity distribution in x-direction.

**Figure 13:**
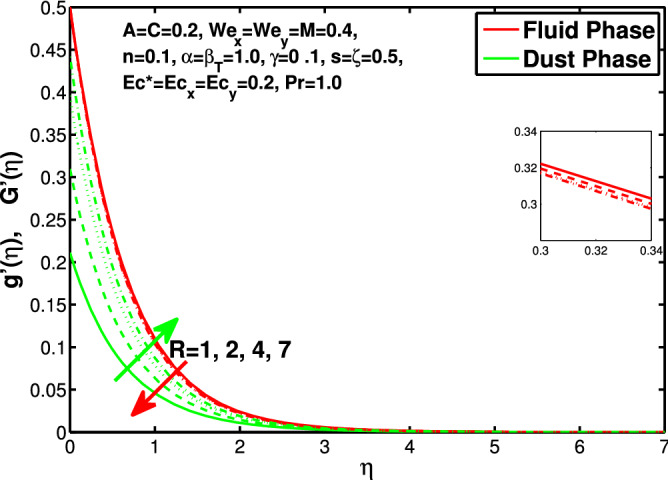
Effect of “*R*” on the velocity distribution in y-direction.

**Figure 14:**
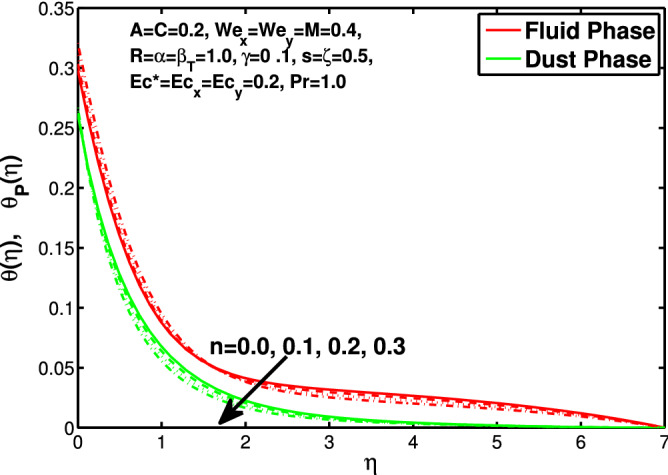
Effect of “*n*” on the temperature distribution.

**Figure 15:**
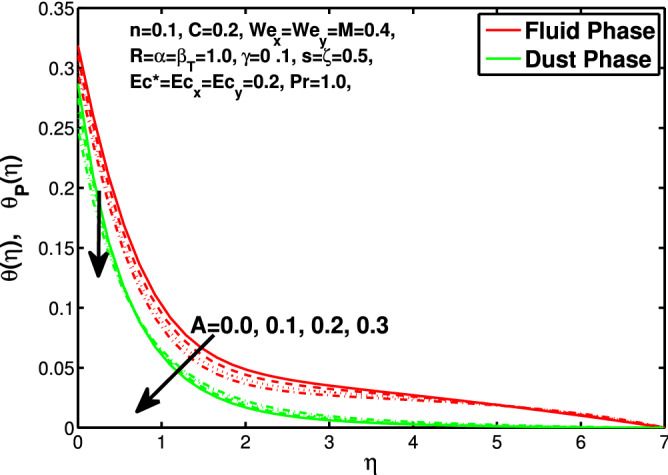
Effect of “*A* on the temperature distribution.

**Figure 16:**
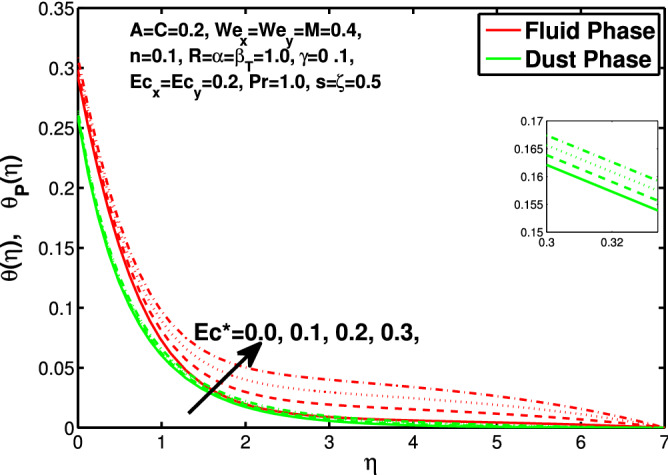
Effect of “$Ec^{*}$” on the temperature distribution.

**Figure 17:**
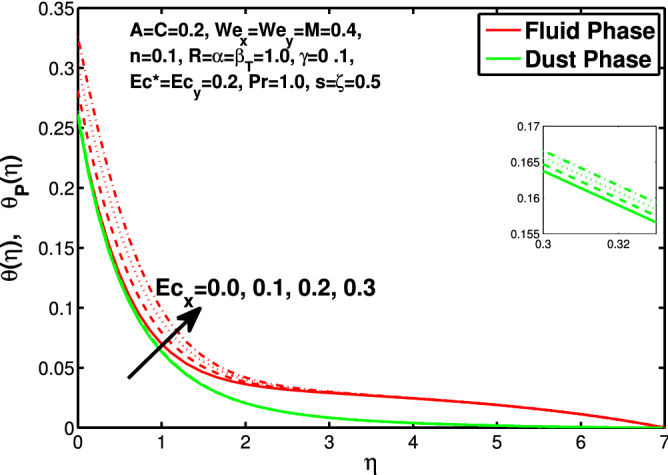
Effect of “$Ec_x$” on the temperature distribution.

**Figure 18:**
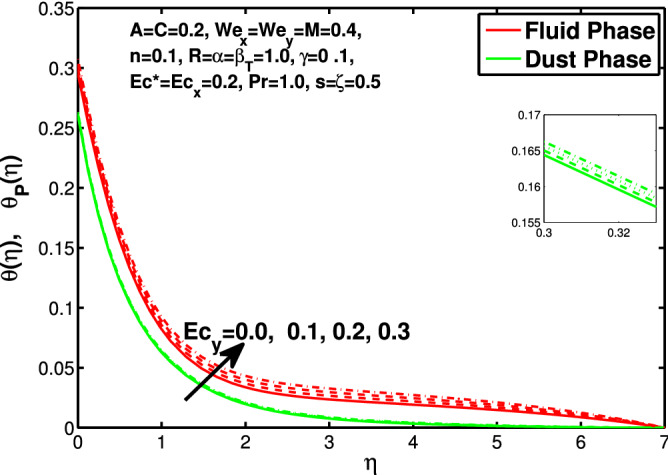
Effect of “$Ec_y$” on the temperature distribution.

**Figure 19:**
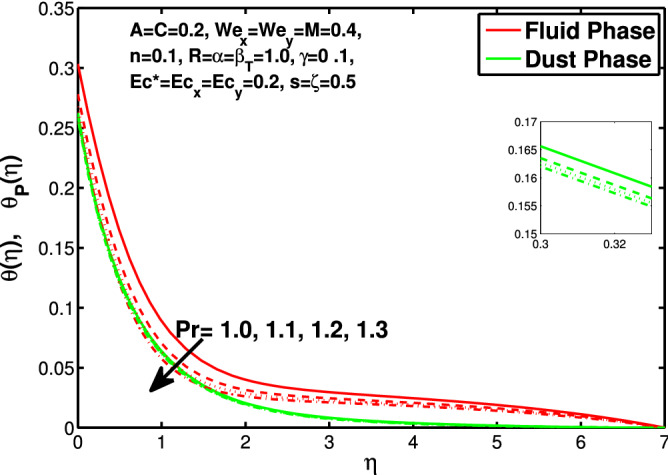
Effect of “*Pr*” on the temperature distribution.

**Figure 20:**
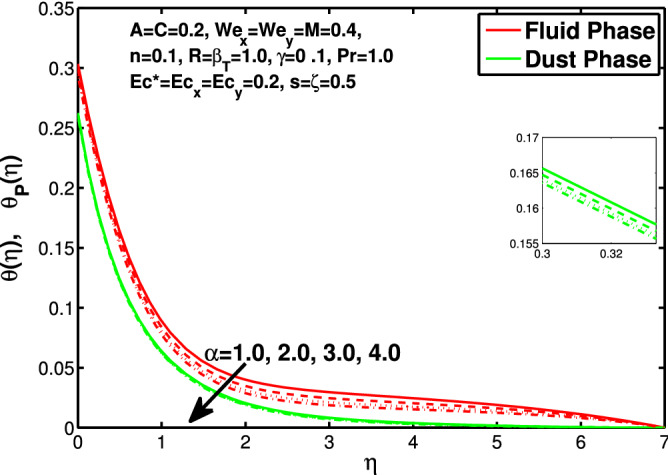
Effect of $\alpha$ on the temperature distribution.

**Figure 21:**
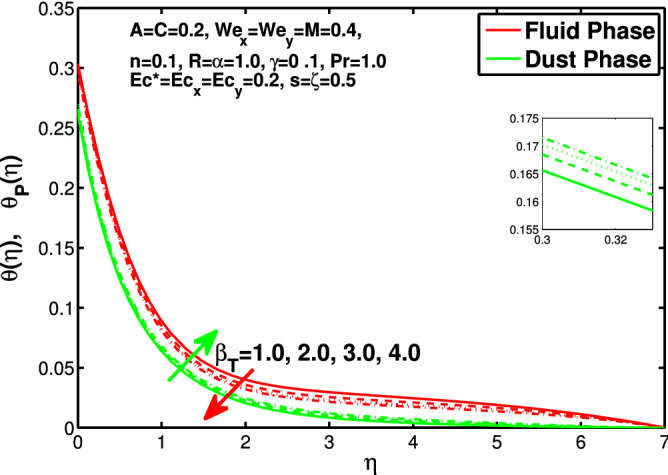
Effect of $\beta _T$ on the temperature distribution.

**Figure 22:**
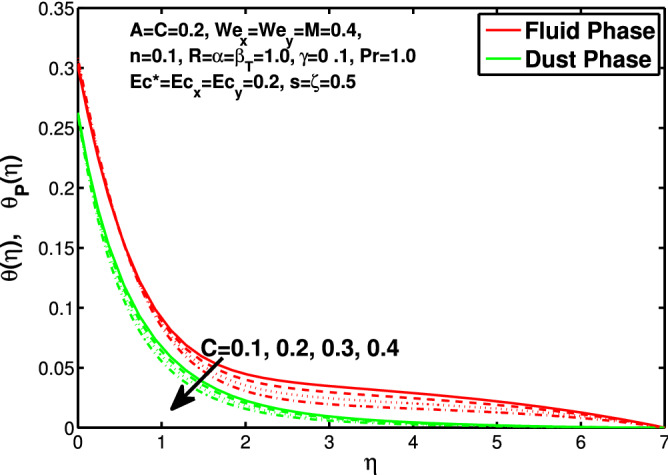
Effect of *C* on the temperature distribution.

**Figure 23:**
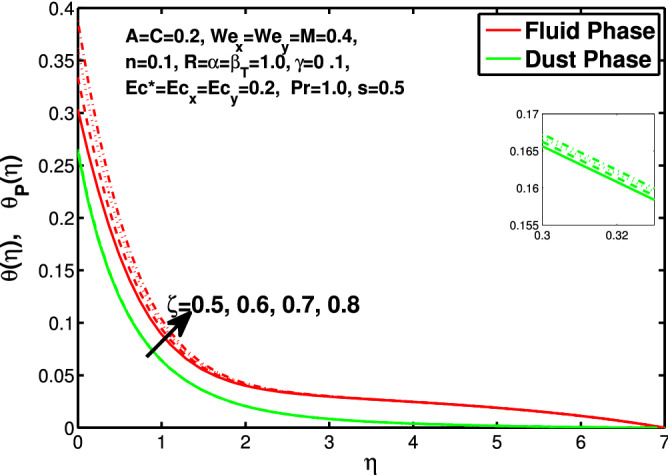
Effect of $\zeta$ on the temperature distribution.

**Figure 24:**
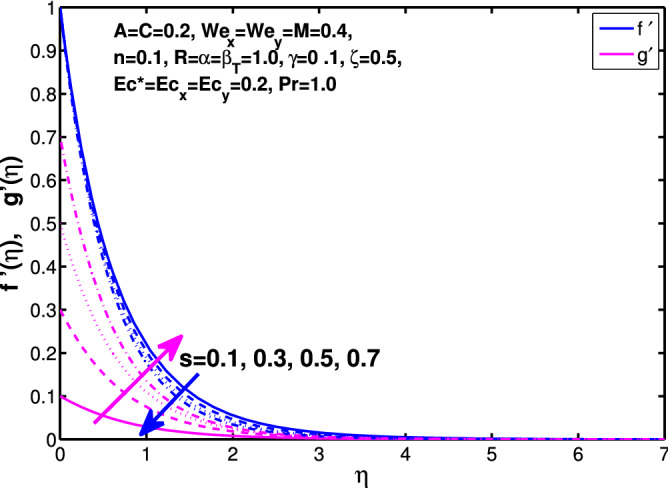
Effect of *s* on the velocity distribution of fluid in x and y-direction.

Figures 10 and 11 depict the outcome of volume fraction that accretion of concentration enhances the resistance to flow due to which boundary layer becomes shorten in both directions. As the flow is solid-liquid so results show that more the solid will redues the flow rate, we can adjust the flow rate by changing the amount of solid particles in mixture flow. Figures 12 and 13 assimilate the out-turn of interaction between fluid and dust particles *R*. The interaction reduces the flow of fluid and enhances the speed of dust particles because more the interaction more the particles will flow but due to interaction the resistance increases due to which speed of fluid reduces. Same results are shown in both directions. Figure 14 shows that while enhancing the power law index there is a decrease in temperature of the fluid. The same results for the velocity profile due to power law index and we know that temperature is connected with kinetic energy relation. So we can say that decrease in velocity will reduce the kinetic energy and in turn the temperature of fluid will decrease. Figure 15 shows that rise of unsteadiness decreases the temperature profile. Enhancement in unsteadiness decline the flow of fluid in turn decreases the temperature of fluid. Figures 16, 17 and 18 show that increment of viscous dissipation parameters $Ec^*$, $Ec_x$ and $Ec_y$ enhances the temperature of the system because heat generated during the dissipation due to viscous forces. The produced heat absorbed by the fluid and thicken the thermal boundary layer of fluid and solid particles. Figure 19 shows the results for Prandtl number. Prandtl number has an inverse relation with the thermal conduction of fluid, the enhancement of *Pr* reduces the temperature of fluid and dust particles as well. Figure 20 shows that enhancement of mass concentration $\alpha$ reduces the temperature profile of fluid after attaining the maximum value for balancing of the temperature profile of the fluid and solid particles. Figure 21 shows that the enhancment of fluid-particle interaction $\beta _T$ reduces the temperature profile of fluid and raises the temperature of solid particles. Figure 22 shows the impact of volume fraction of solid particles *C* on temperature profile. Increase in number of particles the temperature boundary layer decreases because increase in fraction of granules causes resistance results in the generation of internal energy turns to utilized to keep the temperature of the mixture at state of equilibrium. Figure 23 shows that temperature profile increases with increase in Biot number. This is due to the fact that the convective heat exchange at the surface leads to enhance the thermal boundary layer thickness. Figure 24 shows that with the increase in stretching ratio parameter there is decrease in velocity in x-direction and increases in y-direction. And the reason is clear that $s=\frac{b}{a},$ *s* is directly proportional to *b* and inversely proportional to *a*. So increase in *s* increases *b* which is coefficient of $V_w$ and decreases the *a* which is coefficient of $U_w$. In Table 1 we have compared the of results with the online published articles by the Ariel^12^ and Hayat et al.^34^ in which they have solved the problem by exact method and numerical technique as well. The present data founded by bvp4c numerical method comparable with already published results. Tables 2 and 3 exhibit the changes in values of skin friction and Nusselt number for the considered parameters.

**Table 1:** Similarity of values of skin friction coefficient with published data^12,34^ by keeping $n=0$, $M=0$, $A=0$, $R=0$, $We_x=0$ and $We_y=0$.

s	−f”(0)			−g”(0)		
	$Exact^{8} results$	$Approximate^{12} results$	Present findings	$Exact^{8} results$	$Approximate^{12} results$	Present findings
0	1	1	1.000172075	0	0	0.000000013
0.1	1.02025978	1.01952736	1.020660804	0.06684715	0.06684715	0.062427429
0.2	1.03949519	1.03827716	1.040423786	0.14873691	0.15018484	0.134408200
0.3	1.05795478	1.05642139	1.059440011	0.24335980	0.24476799	0.217419213
0.4	1.07578811	1.07406868	1.077719687	0.34920865	0.35039176	0.312560997
0.5	1.09309502	1.09129272	1.095289353	6.46520485	0.46606428	6.420667298
0.6	1.10994694	1.10814635	1.112183080	0.59052892	0.59101139	0.542377495
0.7	1.12639752	1.10814635	1.128437063	0.72453174	0.72460900	0.678186868
0.8	1.14248862	1.14089185	1.144088858	0.86668292	0.86634122	0.828485294
0.9	1.15825383	1.15683905	1.159176650	1.01653870	1.01577291	0.993585147
1.0	1.17372074	1.17253093	1.172544500	1.1737274	1.17253093	1.172020638

**Table 2:** Numerical values of skin friction.

*A*	*n*	$We_x$	$We_y$	*M*	*C*	*R*	*s*	$-f''(0)$	$-g''(0)$	$(1-n) f''(0) + \frac{nWe_{x}}{2} \sqrt{f''(0)^2 + g''(0)}f''(0)^2$	$(1-n) g''(0) + \frac{nWe_{y}}{2} \sqrt{f''(0)^2 + g''(0)}g''(0)^2$
0.0	–	–	–	–	–	–	–	1.2439	0.5322	1.1118	0.4776
0.1	–	–	–	–	–	–	–	1.2742	0.5493	1.1387	0.4929
0.2	0.1	–	–	–	–	–	–	1.3044	0.5664	1.1655	0.5082
–	0.2	–	–	–	–	–	–	1.3927	0.6023	1.0948	0.4782
–	0.3	0.1	0.1	–	–	–	–	1.5036	0.6463	1.0186	0.4461
–	–	0.2	0.2	–	–	–	–	1.5387	0.6516	1.0061	0.4434
–	–	0.3	0.3	–	–	–	–	1.5787	0.6572	0.9929	0.4406
–	–	0.4	0.4	0.1	–	–	–	1.6249	0.6631	0.9790	0.4378
–	–	–	–	0.2	–	–	–	1.6474	0.6743	1.9903	0.4474
–	–	–	–	0.3	–	–	–	1.6845	0.6927	1.0089	0.4561
–	–	–	–	0.4	0.2	–	–	1.7357	0.7177	1.0342	0.4715
–	–	–	–	–	0.3	–	–	1.8242	0.7540	1.0773	0.4937
–	–	–	–	–	0.4	1	–	1.9388	0.8001	1.1316	0.5217
–	–	–	–	–	–	2	–	2.0678	0.8342	1.1909	0.5422
–	–	–	–	–	–	3	–	2.1054	0.8431	1.2078	0.5475
–	–	–	–	–	–	4	0.5	2.1790	0.8548	1.2404	0.5545
–	–	–	–	–	–	–	0.7	2.2077	1.0812	1.2530	0.6867
–	–	–	–	–	–	–	0.9	2.2629	1.5948	1.2768	1.0663

**Table 3:** Numerical values of Nusselt number.

*A*	n	*Pr*	$\alpha$	$\upbeta$	*C*	$\upgamma$	$Ec^*$	$Ec_x$	$Ec_y$	$\zeta$	$-\theta '(0)$
0.0	–	–	–	–	–	–	–	–	–	–	0.3085
0.1	–	–	–	–	–	–	–	–	–	–	0.3194
0.2	0.1	–	–	–	–	–	–	–	–	–	0.3293
–	0.2	–	–	–	–	–	–	–	–	–	0.3088
–	0.3	0.7	–	–	–	–	–	–	–	–	0.2827
–	–	0.8	–	–	–	–	–	–	–	–	0.3211
–	–	0.9	–	–	–	–	–	–	–	–	0.3539
–	–	1.0	1.0	–	–	–	–	–	–	–	0.3825
–	–	–	2.0	–	–	–	–	–	–	–	0.3902
–	–	–	3.0	–	–	–	–	–	–	–	0.3974
–	–	–	4.0	1.0	–	–	–	–	–	–	0.4041
–	–	–	–	2.0	–	–	–	–	–	–	0.4256
–	–	–	–	3.0	–	–	–	–	–	–	0.4421
–	–	–	–	4.0	0.1	–	–	–	–	–	0.4555
–	–	–	–	–	0.2	–	–	–	–	–	0.4984
–	–	–	–	–	0.3	–	–	–	–	–	0.5310
–	–	–	–	–	0.4	0.1	–	–	–	–	0.5577
–	–	–	–	–	–	0.2	–	–	–	–	0.5474
–	–	–	–	–	–	0.3	–	–	–	–	0.5375
–	–	–	–	–	–	0.4	1.0	–	–	–	0.5280
–	–	–	–	–	–	–	2.0	–	–	–	0.5115
–	–	–	–	–	–	–	3.0	–	–	–	0.4949
–	–	–	–	–	–	–	4.0	1.0	–	–	0.4784
–	–	–	–	–	–	–	–	2.0	–	–	0.3669
–	–	–	–	–	–	–	–	3.0	–	–	0.2555
–	–	–	–	–	–	–	–	4.0	1.0	–	0.1441
–	–	–	–	–	–	–	–	–	2.0	–	0.1330
–	–	–	–	–	–	–	–	–	3.0	–	0.1220
–	–	–	–	–	–	–	–	–	4.0	1.0	0.1109
–	–	–	–	–	–	–	–	–	–	2.0	0.1798
–	–	–	–	–	–	–	–	–	–	3.0	0.2267
–	–	–	–	–	–	–	–	–	–	4.0	0.2607

## Concluding remarks

The three dimensional unsteady dusty flow of tangent hyperbolic fluid is studied in this article. As we know that time is really important factor in real world so one of the purpose of this study to check the effects of different variables and factors while keeping in mind the effect of time on flows. Also the three dimensional flows are near to real world problems. Here the used concept is of solid-liquid flows which are of already existing flows on earth and using this concept we can take benefits at industrial level like fluidinzation. How these types of flows can be adjusted according to our requirements by considerering different effects. The effects of magnetic field and viscous dissipation with convection are discussed for fluid and dust particles as well. Also we can adjust the temperature of the system according to our requirments by controlling different used parameters. Outcomes of the current problem are mentioned below. Most of the parameter cause hindrance to flows, let have a glance to the results,

- Increase in power law index, magnetic field, Weissenberg effect, concentration of dust particles, and unsteadiness parameter reduces the flow of fluid and solid granules in both x and y-directions.
- Increase in interaction between fluid and particles drops the flow of fluid, simultaneously increases the velocity (in both x and y-directions) and temperature of dust granules.
- Increase in Prandtl number reduces the temperature of fluid and dust granules as well.
- Increase in viscous dissipation and Biot number rise the temperature of the system, increase the temperature of fluid and dust particles as well.

By using the above results we can control the flow and temerature of the system at industrial level in different areas especially in polymer industry, as in introduction the detailes for the industries and medical field significances are mentioned.
